# Remote Monitoring of Tai Chi Balance Training Interventions in Older Adults Using Wearable Sensors and Machine Learning

**DOI:** 10.21203/rs.3.rs-5389927/v1

**Published:** 2024-12-02

**Authors:** Giulia Corniani, Stefano Sapienza, Gloria Vergara-Diaz, Andrea Valerio, Ashkan Vaziri, Paolo Bonato, Peter Wayne

**Affiliations:** 1 Department of Physical Medicine and Rehabilitation, Harvard Medical School, Spaulding Rehabilitation Hospital, Boston, MA, USA; 2 Luxembourg Centre for Systems Biomedicine (LCSB), University of Luxembourg, Esch-sur-Alzette, Luxembourg; 3 Luxembourg Institute of Health (LIH), Strassen, Luxembourg; 4 Department of Physical Medicine and Rehabilitation, Virgen del Rocio University Hospital, Seville, Spain; 6 BioSensics LLC, Newton, MA, USA; 5 Department of Electronics and Telecommunications, Politecnico di Torino, Torino, Italy.; 7 Osher Center for Integrative Medicine, Harvard Medical School and Brigham and Women’s Hospital, Boston, MA, United States.

## Abstract

Tai Chi, an Asian martial art, is renowned for its health benefits, particularly in promoting healthy aging among older adults, improving balance, and reducing fall risk. However, methodological challenges hinder the objective measurement of adherence to and proficiency in performing a training protocol, critical for health outcomes. This study introduces a framework using wearable sensors and machine learning to monitor Tai Chi training adherence and proficiency. Data were collected from 32 participants with inertial measurement units (IMUs) while performing six Tai Chi movements evaluated and scored for adherence and proficiency by experts.

Our framework comprises a model for identifying the specific Tai Chi movement being performed and a model to assess performance proficiency, both employing Random Forest algorithms and features from IMU signals. The movement identification model achieved high accuracy (micro F1: 90.05%). Proficiency assessment models also achieved high accuracy (mean micro F1: 78.64%).

This study shows the feasibility of using IMUs and machine learning for detailed Tai Chi movement analysis, offering a scalable method for monitoring practice. This approach has the potential to objectively enhance the evaluation of Tai Chi training protocol adherence, learnability, progression in proficiency, and safety in Tai Chi programs, and thus inform training program parameters that are key to achieving optimal clinical outcomes.

## Introduction

Tai Chi, an Asian martial art and mind-body exercise that has gained popularity in the West^1–4^, combines slow, flowing movements with breath training and various cognitive skills, including focused mental attention and imagery^5, 6^. Emerging evidence supports that this holistic practice positively impacts many domains of health highly relevant to older adults, including balance and mobility^7^, musculoskeletal pain^8^, cardiorespiratory function^9^, cognition^10^, sleep^11^, and mood^12^. The benefits of Tai Chi training for fall prevention in older adults are especially robust. Meta-analyses of multiple large-scale trials in both Asia and the West have shown a decrease in fall incidents by 20–45% among practitioners^13–19^. Community-based implementation studies^20^ and cost-effectiveness studies add further support for Tai Chi’s utility in addressing fall-related public health needs^13, 14^.

However, despite this promise, one significant issue limits the translation of these findings into widespread Tai Chi training for fall prevention in older adults. With the exception of some metropolitan areas, the availability of in-person Tai Chi programs is limited. Moreover, for frail and fall-prone older adults, travel to and from such classes includes significant burden and risk, which decreases the likelihood of long-term compliance. An emerging solution for this challenge is home-based virtual instruction. Surveys suggest that adults interested in learning Tai Chi regularly use recorded video and web-based training^1^ despite limited evidence of the effectiveness and safety of these programs^21, 22^. The extension of effective and safe Tai Chi training to the home environment would represent a significant tool for addressing fall risk in older adults. However, novel approaches are needed to objectively monitor performance in the home environment and evaluate training protocol adherence, progression in training proficiency, and safety.

The objective assessment of adherence and performance proficiency represent important methodological challenges for translational Tai Chi research. Whereas dose-response analyses are common and relatively straightforward in pharmacological studies^23^, this causal framework is largely undeveloped in mind-body exercise research. In the specific case of Tai Chi, clinical benefits likely result from three interrelated broad training parameters: duration of practice, sub-components of training regimen practiced (e.g., specific Tai Chi movements or ancillary exercises), and proficiency or quality of performance of sub-component training elements^24, 25^. Recent studies and meta-analyses have highlighted the importance of cumulative practice time on clinical outcomes^26–28^. However, tools for systematically and objectively characterizing training adherence (i.e., which training subcomponents were practiced) and training proficiency (quality of training) have not been developed, and thus, their role in contributing to Tai Chi’s health benefits is not understood. The development of objective tools that characterize both adherence and proficiency could inform optimal training regimens targeting different populations and outcomes, leading to more personalized and patient-centered prescription of Tai Chi practices. Such tools could also be adapted for real-time, actionable feedback, particularly for proficiency, providing practitioners with input to enhance training accuracy and skill acquisition.

**Figure 1.**
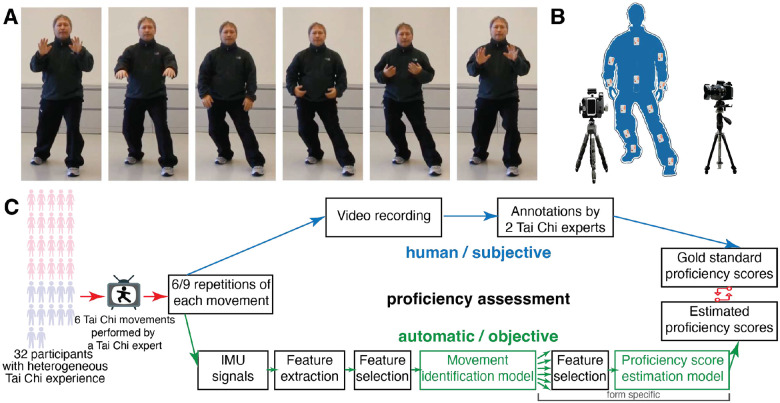
Experimental setup and analysis pipeline **A** Sequence of poses associated with the PUSH movement, illustrating the dynamic movements involved. Sequence of pose for all the other movements is reported in Figure S1. **B** Schematic overview of the data collection setup. Participants performing Tai Chi movements were equipped with thirteen 9-axis Shimmer IMU sensors strategically placed on their upper and lower limbs, as well as on their trunk (with one sensor on the sacrum not depicted). Their movements were captured from both frontal and lateral perspectives using two cameras. **C** Block diagram illustrating the experimental and analytical workflow. Thirty-two elderly participants executed six unique Tai Chi movements. Each movement was assessed and scored by Tai Chi experts. These evaluations were instrumental in both the creation and validation of a model for automatic scoring.

In this context, we developed a framework using wearable sensor data to monitor both training protocol adherence and proficiency among Tai Chi practitioners. Data were collected from 32 participants who performed six traditional Tai Chi movements repeatedly while equipped with 13 inertial measurement units (IMUs) to record detailed motion data. These sessions were video-recorded and subsequently evaluated by two Tai Chi experts to assess the proficiency of each movement, as illustrated in Figure 1. The six Tai Chi movements were based on the Cheng Man Ching Yang Family tradition style^29^, and Tai Chi experts trained within this system visually evaluated proficiency using a formal scoring system.

Our framework utilizes Random Forest (RF) algorithms to analyze features extracted from IMU data. These metrics serve as proxies for Tai Chi proficiency, providing essential indicators of practice content and quality. By analyzing an extensive set of 378 features, we aimed to objectively assess each practitioner’s performance. For evaluating adherence to the training protocol and providing essential indicators of training content, we selected the most relevant features from this set. Similarly, for assessing the physical execution of movements and providing essential indicators of quality, we identified and analyzed different subsets of features, ensuring a comprehensive and accurate evaluation of both aspects (see Figure 1C). Additionally, with this initial study we aim to assess the system’s usability with the intention of refining it further—potentially reducing the number of sensors required—based on insights gained.

By leveraging machine learning and wearable sensor data, our framework bridges the gap between traditional in-person Tai Chi instruction and scalable training that can be performed in a variety of environments. While the technologies employed are well-established^30^, the novelty of our work lies in their specific application to Tai Chi assessment. This combination offers a flexible, adaptable solution for objective, remote monitoring, supporting the broader dissemination of a practice known for its significant health benefits. IMUs provide continuous, precise measurements, making them a ubiquitous solution across diverse environments where other methods may face limitations. Machine learning ensures consistent, scalable evaluations of both adherence and proficiency, making this approach particularly well-suited for Tai Chi practice in diverse settings.

In the following sections, we present the results of our study, detailing the accuracy and reliability of our methodology in evaluating Tai Chi proficiency and adherence among older adults.

## Results

### Movement identification

We employed the features derived from IMU signals to develop an RF classifier trained to discriminate among the six different movements contained in our dataset –i.e., the basis for assessment of adherence to specific training protocol subcomponents. Despite the inherent variability stemming from the execution of each movement in several variations by participants of differing proficiency levels, we posited that the distinct movement patterns and postures intrinsic to each movement should be represented and identifiable in the comprehensive set of 378 features extracted from IMU signals.

**Figure 2.**
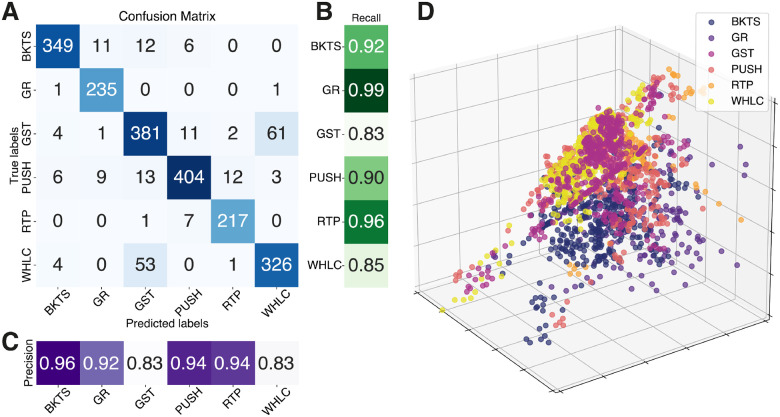
Results of the movement identification model **A** Confusion matrix illustrating the RF classifier’s accuracy in identifying different movements. **B** Recall scores for each movement. **C** Precision scores for each movement. **D** Feature projection into a lower-dimensional space achieved through Sammon mapping, with points color-coded by movement. Acronyms: Raising the Power (RTP), PUSH, Grasp the Sparrow’s Tail (GST), Wave Hands Like Clouds (WHLC), Brush Knee Twist Step (BKTS), Golden Rooster (GR)

The RF classifier achieved an average micro F1 score of 90.05 ± 0.35% in identifying the six Tai Chi movements, demonstrating the capability of IMU data to capture movement patterns specific to each movement accurately. The minimum redundancy maximum relevance (mRMR) algorithm selected a set of 15 features as the optimal ones for this classification task. The confusion matrix associated with the classifier’s predictions in Fig 2A displays strong diagonal values, indicating high accuracy in correctly identifying each movement. In terms of precision and recall, the classifier demonstrated high performance across most movements, with precision values ranging from 83% to 96% and recall values from 83% to 99% (Fig 2B and C), highlighting some variability in the classifier’s performance across different movements.

The movement Golden Rooster (GR), for example, shows particularly strong recognition with 235 correct movements identified out of 237, along with high precision and recall values of 99% and 92%, respectively. This indicates that the classifier not only correctly identified the majority of GR instances but also did so with few false positives, reflecting its robustness in recognizing this movement. Conversely, the movement Grasp The Sparrow’s Tail (GST) had a precision of 83%, with 61 instances being incorrectly identified as Wave the Hands Like Clouds (WHLC). This suggests that there is room for improvement in reducing the number of false positives for GST, as well as enhancing the classifier’s ability to distinguish between movements with similar movement patterns.

It is worth noticing that a relevant portion of most of the misclassifications was ‘symmetric,’ meaning that 61 of GST trials were classified as WHLC and, vice versa, 53 of WHLC were classified as GST. This supports the idea that the model is generally strong, but some movements are similar or have similar characteristics, making it difficult to distinguish between them. Indeed, these two movements share common motion patterns (see Figure S1); for example, in both WHLC and GST, the hands are lowered from shoulder height with trunk torsion and circular motion while slightly bending the knees. Such similarities can cause the classifier to confuse the movements, highlighting the need for more refined feature extraction or additional contextual information to improve classification accuracy.

Furthermore, our analysis revealed that out of 219 misclassified movements, 115 were assessed by the Tai Chi master as having the lowest proficiency level. This suggests that our movement identification model struggles to differentiate between movements when they are executed with lower proficiency. While this result aligns with expectations, it highlights the necessity of distinguishing movements regardless of skill level to provide consistent and accurate feedback to users. Therefore, future work should concentrate on identifying features that enable the reliable differentiation of Tai Chi movements, independent of proficiency level.

The feature projection plot in Figure 2D provides visual insight into the spatial distribution of each Tai Chi movement in a reduced feature space. The notable overlaps observed between several movements, such as GST and WHLS, or PUSH and other movements, can be largely attributed to the inherent similarities in the motion across different Tai Chi movements. Nevertheless, some clustering is evident, particularly with movements like BKTS, where distinct groupings in the feature space are visible. This clustering reflects that despite the similarities in movements, the features extracted from IMU data are capable of capturing enough distinct movement characteristics to effectively distinguish between most movements.

Overall, this initial exploration demonstrates the feasibility of using IMU data for detailed movement analysis in Tai Chi, specifically in identifying the movements being performed. This represents the first step in the cascade for building the hierarchical model outlined in Figure 1C. At this stage, we have primarily shown the ability to build a classification algorithm for identifying the movements, paving the way for the next steps in assessing proficiency levels associated with each movement.

### Proficiency assessment

After establishing a model to identify the Tai Chi movement using IMU-extracted features, we developed six models, each to assess the proficiency of practitioners during the performance of one movement. This approach entailed selecting relevant features and conducting individualized training for each model based on the data captured during the execution of each movement.

To ensure the best features were selected for our task, we employed the mRMR algorithm before training each model. The features were ranked using this algorithm, and then only the minimum number necessary to achieve 95% of the accuracy obtained with the full set of features was selected. Through this implementation, a varied number of features were identified for each model, ranging from four to ten. All the features selected for each movement are reported in the Supplementary Table S1.

The effectiveness of the models was assessed using the leave-one subject-out cross-validation and two distinct F1 scores to measure assessment accuracy. The first score, termed the “repetitions F1 score,” evaluates the precision of the models in classifying each individual repetition of a movement performed by a practitioner. The second measure, the “overall F1 score,” was derived using a method that mirrors the scoring approach of Tai Chi experts in contemporary competition (see Methods). Specifically, Tai Chi experts assess all repetitions of the same movement from one practitioner collectively and assign a single comprehensive score to the entire set of repetitions. To simulate this expert evaluation, the overall F1 score aggregates all repetitions performed by a subject for each movement and assigns the most frequently assessed score by the RF among these repetitions as the final score for that movement.

**Figure 3.**
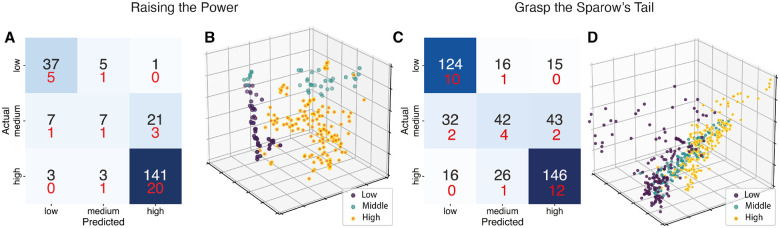
Results of the proficiency assessment models **A, C** Confusion matrix illustrates the RF classifier’s accuracy in a 3-class classification task for ‘Raising the Power’ (A) and ‘Grasp the Sparrow’s Tail’(B) movements. Repetition classification numbers are shown above, and overall task classification numbers are below (in red). **B, D** Feature projection into a lower-dimensional space is achieved through Sammon mapping, with points color-coded by class for the ‘Raising the Power’ (B) and ‘Grasp the Sparrow’s Tail’ (D) movements.

The data presented in Table 1 and in Figures 3 and S2 reveal significant variations in the accuracy of these models across different Tai Chi movements both in the repetitions micro F1 score (mean repetitions micro F1: 72.97%, min: 64.93%, max: 80.98%) and in the overall micro F1 score (mean overall micro F1: 78.64%, min: 72.69%, max: 83.90%).

A notable example is RTP, which, utilizing eight features, achieved remarkable accuracy (see Figure 3B. Repetitions micro F1 score: 80.98 ± 1.53; overall micro F1 score: 83.90 ± 2.48). As illustrated in Figure 3B, a features projection in a three-dimensional space obtained through Sammon mapping demonstrates effective clustering of the three proficiency classes. The model exhibited excellent sensitivity for the low (recall = 86%) and high (recall = 95%) classes, showcasing its strong capability in accurately identifying instances within these categories. In contrast, the sensitivity for the medium class was lower (recall = 20%), revealing a significant challenge in identifying medium class instances accurately, which could be attributed to the lower number of samples in this class.

Another behavior worth noting emerged in the GST analysis (see Figure 3C and D). Interestingly, in this movement, the RF model’s performance in classifying individual repetitions was found to be moderate (repetition F1 score = 67.96%), yet the overall micro F1 score significantly increased to 81.41%. This suggests high variability across repetitions, but a majority still accurately reflected the scores assigned by the Tai Chi expert. Consequently, despite numerous errors in individual repetitions, the model proficiently assesses each subject’s final score, underscoring its effectiveness in overall assessment despite challenges in repetition-level precision.

Overall, the development and evaluation of six distinct models tailored to specific Tai Chi movements have demonstrated a range of accuracies, from moderately good to good across the movements. This varied performance underscores the complexity of modeling Tai Chi, where the effectiveness of each model depends not only on the selected features but also on the inherent variability and technical demands of each movement.

To evaluate the robustness of our proficiency assessment model against misclassified movements, we investigated the impact of these errors on the accuracy of proficiency predictions. For instance, we examined whether a GST movement sample incorrectly labeled as WHLC could still be accurately assessed by the WHLC proficiency model. This analysis aimed to determine the resilience of our hierarchical model to initial misclassification errors. We utilized the models originally trained for each movement proficiency assessment and tested them on the misclassified samples from the movement identification model. Our analysis revealed a decrease in overall proficiency assessment accuracy, with proficiency being correctly assessed in 80 out of the 219 samples misclassified by the movement identification model. Notably, there was significant variability in the results. For example, the proficiency assessment model for the RTP movement correctly classified 15 out of 15 samples misclassified as RTP by the movement identification model. In contrast, the GR model correctly assessed proficiency in 2 out of 20 samples. These findings suggest that while our model exhibits some robustness, particularly in certain movement-specific models, there is considerable variability depending on the specific movement.

Finally, we trained an RF classifier to assess Tai Chi proficiency across all movements, testing a unified model based on the hypothesis that common characteristics like movement smoothness or execution confidence could indicate proficiency despite the unique biomechanics of the six movements. We aimed to determine if these universal attributes could reliably assess proficiency across different Tai Chi movements and overcome the potentially lowered performance on misclassified movements by the first module of the hierarchical cascade. The unified model’s repetitions F1 score was 61.71 ± 0.53%, with an overall F1 score of 65.80 ± 0.91%. The confusion matrix and feature projection for this model are reported in Figure S3.

Our findings indicate that the movement-specific models demonstrated superior accuracy compared to a unified model. By tailoring each model to the unique characteristics of individual Tai Chi movements, we achieved more precise proficiency assessments. However, there is still a need to improve the robustness of our models against initial movement misclassification. Enhancing this aspect will be crucial for achieving consistent and accurate evaluations across all movements.

## Discussion

Among community-living older adults, falls are the leading cause of serious injury, disability, nursing home placement, and injury-related death^31–34^. Fatal falls in older persons exceed the death rate from the opioid epidemic by a factor of four^35, 36^. Even non-traumatic falls can lead to fear of falling, reduced physical activity, psychosocial dysfunction, and loss of autonomy^37–40^. The high incidence and long-term effects of falls among older adults result in substantial medical costs to individuals and society^41^. Exercise is considered one of the best strategies for reducing fall risk, and among exercise modalities, Tai Chi is one of the best-evidenced options^14^. The US Centers for Disease Control and Prevention and many other national organizations endorse Tai Chi for fall prevention and overall health and rehabilitation^42^. However, utilization of Tai Chi remains very low, with surveys suggesting only 1.2% of the entire adult population uses Tai Chi for health^43^. Limited access to Tai Chi programs - particularly for individuals who live in rural areas and underserved communities - has hampered the potential societal impact of such programs. Paralleling broader trends in telemedicine^44^, an increasing number of platforms are currently available to deliver Tai Chi via DVDs and online websites. However, none of these platforms provide tools for monitoring adherence and proficiency to Tai Chi training, which is essential for monitoring program effectiveness and safety.

In this study, we introduce a framework for evaluating Tai Chi adherence and proficiency using a RF algorithm trained on features derived from IMU signals. While machine learning and feature engineering are established methods, the strength of our work lies in their focused application to Tai Chi assessment. This approach not only enhances the monitoring and evaluation of Tai Chi practice but also supports the broader dissemination of a discipline already recognized for its significant health benefits. Our research was conducted in two primary phases: first, we developed a model to identify the specific Tai Chi movement being performed; second, we created a model to assess the proficiency of practitioners in each movement. Overall, the framework showed solid accuracy in identifying the performed Tai Chi movement and good performances in assessing the proficiency scores of practitioners.

In light of the advancements made and the recognition that further refinement is necessary, it is important to consider the broader implications of the developed framework, particularly its potential to positively impact our understanding of the health benefits of Tai Chi practice and its broader-scale implementation. A large and growing body of studies has firmly established that consistent engagement in Tai Chi yields a broad range of health benefits^6, 7, 9–12, 18, 45^, with particularly large and consistent benefits related to fall prevention^46–48^. However, the health and other functional benefits of Tai Chi likely stem from more than just the quantity of time allocated to training but also from the quality (i.e., proficiency) of training. Along with time stamps for duration adherence to each training element, the proficiency metrics proposed in this study could be used to better develop ‘dose-response’ relationships between training and health outcomes, integrating metrics of both quantity and quality of training. Specific components of proficiency might also provide insights into the biomechanical and physiological mechanisms underlying Tai Chi’s health benefits. Longitudinal metrics of proficiency might also be used as feedback to guide individual practitioners, as well as information for Tai Chi instructors to identify variability in students’ learning trajectories. The use of IMU-based proficiency metrics also represents a potentially useful tool for monitoring home-based virtual Tai Chi training, which increases access to training across more diverse geographic and socioeconomic populations. This remote monitoring capability, enabled by wearable sensors, addresses one of the grand challenges in bridging societal needs for human health care with the development of novel technologies as identified by a group of key opinion leaders^49^.

Utilizing IMU technology for this purpose offers the advantage of ubiquity; the process is subject-independent and location-independent, meaning it can be conducted with any practitioner in any setting. This flexibility makes the framework highly adaptable and scalable, suitable for diverse environments without the need for specialized facilities. Another option for proficiency assessment could be using video recordings, leveraging the fast-growing capabilities of human pose estimation techniques^50^. This method has the benefit of not requiring any special instrumentation, as the session can be recorded with any smartphone. However, video-based assessments have drawbacks, such as being impractical in crowded group classes or poorly lit rooms, which can significantly affect the accuracy of the pose estimation. Therefore, the main advantage of our IMU-based system is its complete ubiquity, providing consistent and reliable assessments regardless of the environment^51^.

More broadly, the key insight from our study is that while IMU data effectively captures essential motion characteristics for movement scoring, it is imperative to construct a comprehensive dataset that encompasses all proficiency levels and accurately reflects the wide range of performance variability. Furthermore, our results suggested that tailoring the model to the specific characteristics of each movement is critical for enhancing the accuracy of proficiency evaluations. This involves adapting the model to closely align with the detailed phases and unique aspects of each physical exercise, thereby improving its capability to accurately assess proficiency.

Building on this understanding, integrating IMU technology with ML algorithms offers an objective framework for assessing Tai Chi proficiency. This method provides consistent, quantifiable performance measures, avoiding the variability of traditional subjective evaluations. The RF classifier achieved a micro F1 score of 90.05 ± 0.35% in distinguishing six Tai Chi movements. The confusion matrix shows strong diagonal values, indicating high accuracy, though some misclassifications suggest areas for improvement. This result confirms the effectiveness of IMU data in capturing the unique movement dynamics of each movement, providing a basis for assessing proficiency levels. This finding is consistent with previous literature^52^, where substantial efforts have been dedicated to activity recognition utilizing IMU data, including the identification of various tasks in physical activities scenarios^53, 54^.

Accurate identification of Tai Chi movements is critical for two primary reasons. First, it allows monitoring adherence by confirming that the practitioner is performing the intended movement and accurately identifying which movement is being executed. Second, once the Tai Chi movement is correctly identified, this information is crucial for channeling data to the correct movement-specific model for proficiency assessment. In our study, we showed that misclassification in the movement classification hindered the overall accuracy of the proficiency assessment models, and, at the same time, the unified model is less capable of accurately assessing proficiency. Effective differentiation between movements allows for targeted and relevant proficiency assessments, directly addressing the unique requirements of each Tai Chi movement. This hierarchical approach enhances the overall efficacy of the evaluation framework, providing a comprehensive method for assessing both adherence and proficiency in Tai Chi practice.

Movement-specific models in our study yielded good micro F1 scores across different Tai Chi movements. The dataset includes movements with varying difficulty levels, posing challenges for both expert and model-based proficiency assessments. Simpler movements like “Raising the Power” show less performance variability and are easier to model and assess accurately. In contrast, complex movements such as “Grasp the Sparrow’s Tail” exhibit greater variability, challenging the scoring methodology and the model’s accuracy in learning and mimicking the expert’s approach.

To further enhance proficiency classification performance, a nuanced feature extraction approach should account for the distinct phases and movements of each Tai Chi form. While standard IMU signal features broadly indicated Tai Chi movements, a detailed strategy distinguishing movement phases could provide deeper insights. , reducing complexity and cost. Additionally, exploring end-to-end modeling strategies could improve classification by learning directly from raw data, reducing human bias, and potentially identifying new critical features^55^.

A key consideration in this study is the methodology for ground truth assignment. Tai Chi experts evaluated all repetitions by a subject collectively, assigning a single comprehensive score. This approach fails to capture variability across repetitions. For example, if a practitioner performs three out of four repetitions well but one poorly, the final score does not reflect this variability, potentially misrepresenting proficiency. This aggregated scoring method introduces inaccuracies in the ground truth for training. Future work could benefit from a more granular approach, evaluating and scoring each repetition individually for a more accurate representation of performance.

In our study, we utilized a full-body sensor setup to ensure comprehensive coverage of all movements involved in Tai Chi practice. This approach allowed us to capture a wide range of kinematic data from multiple regions of the body. Upon analyzing the data, we found that certain sensors were more informative than others. The mRMR algorithm highlighted pivotal roles for the right ankle, chest, both shanks, and right wrist, reflecting Tai Chi’s full-body coordination. Sensors on the right side of the body, such as the right wrist and right shank, were crucial, whereas those on the back, left ankle, and right arm were less informative, suggesting fewer sensors could suffice. Based on these findings, we anticipate that in future iterations of the system, we could reduce the number of sensors without compromising accuracy. This would simplify the setup and make it more cost-effective, aligning with our goal of making Tai Chi monitoring systems more accessible and user-friendly.

The proposed framework lays the foundation for using machine learning and wearable technology to track adherence and assess performance proficiency, enhancing monitoring of progress and safety. By reducing the need for specialized facilities and personnel, this approach has the potential to democratize access to Tai Chi, making it more accessible to a broader audience. Additionally, our adaptable framework can be extended to other movement-based practices such as physical therapy, yoga, and strength training, potentially improving health outcomes through personalized, data-driven feedback. This approach holds significant promise in the field, offering detailed, personalized assessments that cater to individual needs and making the process of movement evaluation more objective and data-driven.

## Materials and methods

### Participants

The study was approved by the Institutional Review Board at Mass General Brigham (protocol number 2018P001218) and all experiments were performed in accordance with relevant guidelines and regulations. The human images in Figure 1 and S1 represent study personnel and were collected solely for illustrative purposes. Informed consent was obtained for the publication of these identifying images in an online open-access format, in compliance with requirements for publicly accessible research. The study included 32 healthy participants aged 60 to 85 years (20 females, mean age 70.20 ± 4.88) to represent a range of Tai Chi experiences. The inclusion criteria for the study were based on age (60 to 85 years) and the ability to ambulate for 15 minutes without the need for an assistive device. The exclusion criteria included chronic neuromuscular conditions, hospitalization for acute medical conditions in the six months preceding the study, active cancer, chronic use of pain medications, or cognitive impairment as indicated by a Mini-Mental State Examination score of less than 24. Participants were screened and provided informed consent.

### Tai Chi protocol

Each eligible participant was asked to perform 6 Tai Chi movements based on the Cheng Man Ching style^29^, namely *Raising the Power (RTP), PUSH, Grasp the Sparrow’s Tail (GST), Wave Hands Like Clouds (WHLC), Brush Knee Twist Step (BKTS), Golden Rooster (GR)*. These movements were selected because they represent classical exercises that are well-suited for individuals with no prior experience in Tai Chi, while still incorporating a wide range of movements, particularly beneficial for balance training. Before commencing each movement, participants were presented with a video modeling the specific task, as performed by a Tai Chi expert with over 35 years of training experience. Participants were afforded the opportunity to watch the video repeatedly until they felt confident in their ability to perform the movement independently. Upon readiness, the video playback ceased, and participants were asked to execute the movement for a specified number of repetitions, which varied depending on the specific movement – either 6 or 9 repetitions, depending on the movement. PUSH, GST, BKTS, WHLC, and GR were practiced bilaterally, with participants performing the movement both with their right and left foot forward (sequence decided randomly). In contrast, the RTP movement in the protocol, which involves symmetrical movements performed with both sides of the body, was executed in just one variation. Participants were allowed to cut short the practice or avoid a movement if they didn’t feel comfortable performing it. Overall it resulted in a total of 2131 repetitions (225 for RTP, 460 for GST, 237 for GR, 447 for PUSH, 378 for BKTS, 384 for WHLC) that were included in the analysis.

### Data collection

Thirteen Shimmer3 (Sensing Health with Intelligence, Modularity, Mobility, and Experimental Reusability) devices (Shimmer Research, Dublin, Ireland), each equipped with a 9-axis inertial measurement unit featuring a tri-axial accelerometer, gyroscope, and magnetometer, were employed to capture movement and gather comprehensive kinematic data throughout the sessions. The sensors were calibrated against parameters used by the LEGSys^™^ (BioSensics, MA, USA). Specifically, the sensor ranges were constrained to ±4 g for the accelerometer, ±2000 deg/s for the gyroscope, and ±1.9 G for the magnetometer, with a sampling frequency established at 100 Hz. Once initialized and synchronized, these devices were fixed to the participant’s body using elastic belts and adhesive tape, providing secure placement on the predetermined body segments for data collection. The placement of the units, as indicated in Figure 1B, was bilateral, covering both the right and left sides of the body at locations including the chest, upper arm, wrist, abdomen, back, thigh, shank, and ankle ensuring a comprehensive movement analysis. To mark the beginning and conclusion of each movement within the dataset, an event marker was utilized via the Shimmer software, which was also used to configure device settings and manage the data after collection. In addition to the kinematic data, each Tai Chi session was recorded using two GoPro cameras (GoPro, Inc. San Mateo, CA, USA) to capture frontal and lateral views. This provided a visual reference to accompany the data, allowing for a comprehensive assessment and scoring of the performance by the Tai Chi expert.

### Movement scoring

Two Tai Chi experts evaluated each participant’s performance using both frontal and lateral video recordings. They assessed all six movements in the study protocol, including bilateral variation and repetition. A multi-item proficiency scoring metric was developed using a modified Delphi consensus process. The ground truth for performance proficiency was derived by establishing a scoring system based on criteria similar to those used in Tai Chi competitions and other similar disciplines, such as figure skating.

The scoring instrument underwent a semi-formal validation process which included: 1) A review of existing academic literature and scoring instruments used in international competitions; 2) Drafting of an initial scoring instrument and protocol; 3) Formation of an expert panel including 6 Tai Chi practitioners with an minimum of 20 years experience and 4 experts in biomotion and engineering who independently assessed the initial scoring protocol; 4) An in-person group discussion addressing expressed limitations of the instrument’s first draft; and 5) Development of a revised scoring instrument integrating feedback from expert panel. The final scoring instrument included the following criteria to evaluate the performance: gross competency in choreography, clear expression of Yin/Yang, alignment and posture, dynamic integration, and range of motion. These criteria were then employed by two Tai Chi experts who independently reviewed video recordings. Inter-rater reliability between experts in their scoring assessments was high (intraclass correlation of scores of 0.99).

**Figure 4.**
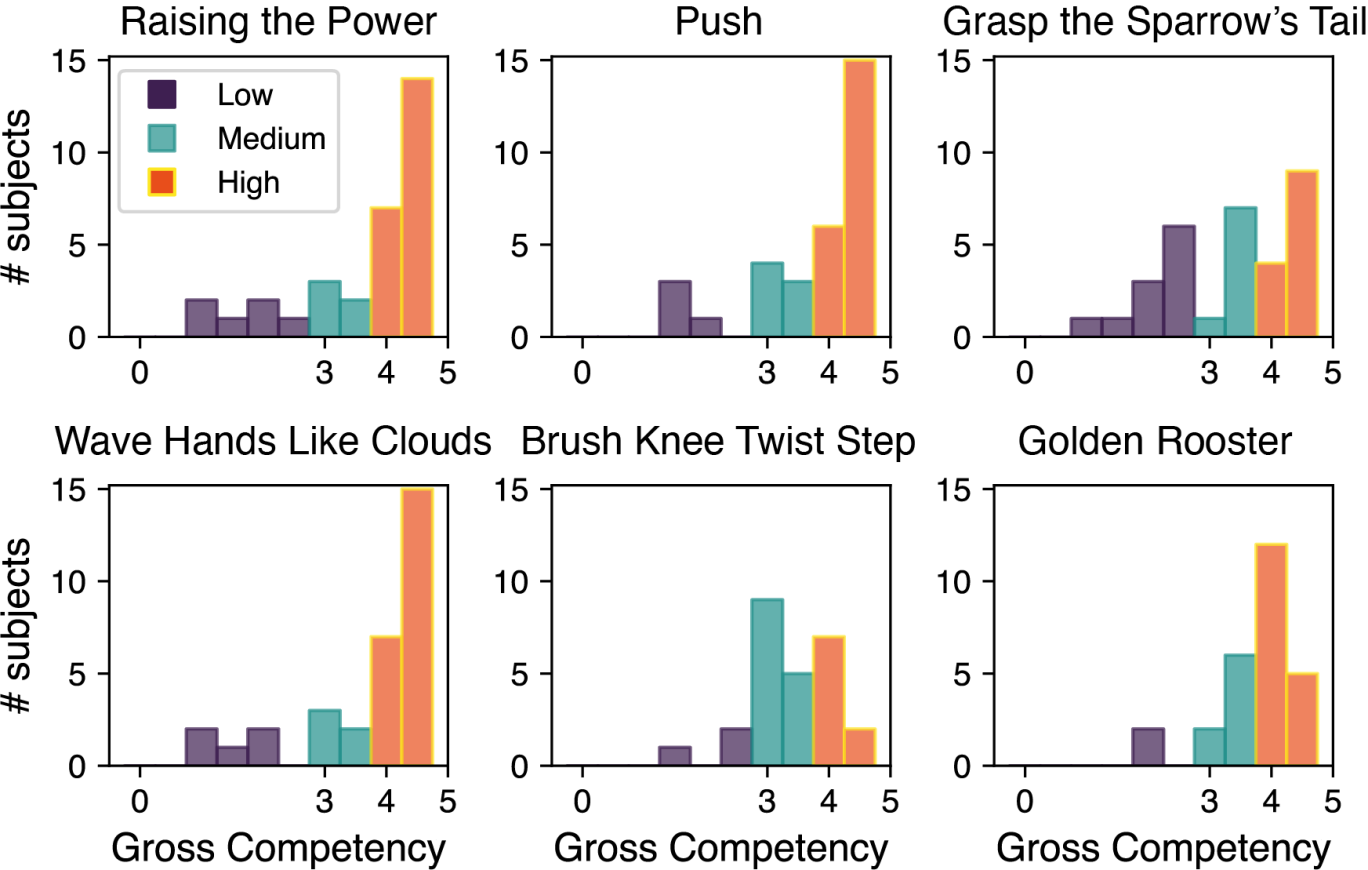
Distribution of the gross competency score among subjects for the six Tai Chi movements in the proposed protocol. The original ten-level scoring system, ranging from 0 to 5 with 0.5 increments, was consolidated into three proficiency classes: low, medium, and high.

In this study, we limited the assessment of proficiency to a single measure of overall choreographical execution accuracy (gross competency). This feature was scored from 0 to 5, with a granularity of 0.5, allowing for a nuanced assessment. For the sake of the analysis presented in this manuscript, we further simplified the scoring method. Instead of using the ten-level Gross Competency scale, we categorized scores into three proficiency levels: below 3 as low, between 3 and 4 as medium, and 4 and above as high. This modification improved our analysis efficiency by mitigating class imbalances and sample size limitations (see Figure 4). Given the limited data available and the natural distribution of the classes, no further balancing techniques were applied to avoid distorting the data distribution or introducing artifacts, ensuring that the analysis remained as close as possible to the original data characteristics.

### Data preparation and preprocessing

Each signal from the IMU devices was initially digitally resampled to a frequency of 32 Hz. Following this, two distinct filtered time series were produced using a 4th-order Chebyshev filter configured for low pass and band pass filtering. These derived time series contain correlated yet distinct information; the low pass filtered signal is better suited for capturing static orientation and alignment, while the band pass filtered signal more accurately reflects dynamic movement characteristics. The high pass filter was set with a cut-off frequency of 0.1 Hz to eliminate the DC component, and the low pass filter was set to 4 Hz. This upper-frequency limit was chosen after an analysis of the frequency spectra, which indicated no significant signal content above this threshold.

The processed signals were then segmented into individual movements using digital markers placed by technicians during data collection. Further segmentation into repetitions was performed using an automatic algorithm that identified velocity zero crossings, which correspond to the brief pauses subjects took between the end of one movement sequence and the start of the next. The accuracy of this segmentation into repetitions was manually confirmed and adjusted by the research team to ensure precise labeling and to avoid errors in data categorization.

### Feature Extraction and selection

For each repetition, sensor, and axis, key features were extracted, such as dominant and mean frequencies in both bandpass and lowpass filtered signals, and orientation metrics, along with the cross-correlation between sensors placed on the ankle and wrist to measure the synchrony of limb movements. The analysis resulted in a comprehensive set of 378 features derived from 13 IMU sensors.

The process of feature selection utilized the Minimum Redundancy Maximum Relevance (mRMR) algorithm^56^. This method assesses each feature’s predictive ability for the target variable and aims to reduce overlap between features. Using mRMR, features were prioritized based on their significance, ensuring focus on the most impactful ones. Subsequently, a predictive model was trained with an increasing number of features, from 1 to 378, chosen based on the ranking provided by the mRMR method. The best group of features for each predictive model tested was selected as the one with the minimum number of elements reaching an accuracy of at least 95% of the performance reached when using the full set of features (see Figure S3). This approach ensured a balance between model complexity and predictive accuracy, leading to an efficient and effective feature set for the analysis.

### Predictive model

The predictive model employed was the Random Forest algorithm^57^, selected for its proficiency with high-dimensional data and resistance to overfitting. This approach is particularly suitable for small datasets, as demonstrated in previous studies^58, 59^. The model was trained with 100 trees and out-of-bag prediction for accuracy estimation.

Performance evaluation used a leave-one-subject-out cross-validation strategy, training the model on all but one subject, then testing on the held-out subject, repeated for each subject and 20 times in total. Repeating the process 20 times ensures robustness and stability, accounting for variability in performance and providing a more reliable evaluation. This approach was chosen to avoid overfitting to specific subjects, given the limited number of 32 subjects, ensuring the model generalizes well to unseen individuals and making better use of the available data compared to a standard train-test split.

In the initial phase, each repetition of a movement by a subject was considered a unique data point, significantly increasing the dataset’s size and diversity. Instead of a standard 32 data points per movement, we expanded this by factoring in the number of repetitions and variations for each of the 32 subjects. This method enhanced the dataset’s variability, improving the model’s learning potential. However, the original scoring system adopted by the Tai Chi experts averaged the scores of all repetitions for each subject, assigning a single score per movement. To mirror this in our assessment process, as a second step, we aggregated the scores assigned for each repetition for each subject, selecting the most voted score across these repetitions as the final score for each movement per subject.

The model’s performance was assessed using the micro F1 score. For each iteration of the cross-validation, the F1 score was computed, with the average score across all iterations serving as the final performance metric.

## Data availabilty

Sensor data used in this study are available from the corresponding authors upon reasonable request. Code is available at https://github.com/gcorniani/TaiChi_sensor.

## Figures and Tables

**Table 1.** Models performance in assessing Tai Chi proficiency Performance of the RF classifier in assessing single repetition and overall gross competency scores in Tai Chi for the movement-specific models. The repetitions micro F1 score was calculated based on the score assigned to each individual repetition of the movements, while the overall micro F1 score was obtained by assigning a single, majority-vote-based score for each participant for each movement, reflecting the approach of the Tai Chi experts.

Movement	# Features Selected	Repetitions micro F1	Overall micro F1
Raising the Power	8	80.98 ± 1.53	83.90 ± 2.48
Push	8	71.12 ± 1.12	74.37 ± 2.34
Grasp the Sparrow’s Tail	5	67.96 ± 0.83	81.41 ± 3.35
Wave Hands Like Clouds	10	77.83 ± 0.73	81.87 ± 1.88
Brush Knee Twist Step	4	64.93 ± 1.10	72.69 ± 2.95
Golden Rooster	6	75.00 ± 1.37	77.59 ± 2.48
